# Complete Sequence of the Smallest Polyomavirus Genome, Giant Guitarfish (*Rhynchobatus djiddensis*) Polyomavirus 1

**DOI:** 10.1128/genomeA.00391-16

**Published:** 2016-05-19

**Authors:** Jennifer A. Dill, Terry F. F. Ng, Alvin C. Camus

**Affiliations:** a Department of Pathology, University of Georgia, Athens, Georgia, USA; b Division of Viral Diseases, National Center for Immunization and Respiratory Diseases, Centers for Disease Control and Prevention, Atlanta, Georgia, USA

## Abstract

Polyomaviruses are known to infect mammals and birds. Deep sequencing and metagenomic analysis identified the first polyomavirus from a cartilaginous fish, the giant guitarfish (*Rhynchobatus djiddensis*). Giant guitarfish polyomavirus 1 (GfPyV1) has typical polyomavirus genome organization, but is the smallest polyomavirus genome (3.96 kb) described to date.

## GENOME ANNOUNCEMENT

Polyomaviruses have been found in a range of avian and mammalian species. Although some persist asymptomatically, other polyomavirus species cause diseases ranging from urinary tract hemorrhage to neoplasia (1–5). Historically, taxonomic classification included three genera, the *Orthopolyomavirus* and *Wukipolyomavirus* from mammals and *Avipolyomavirus* from birds (6, 7). However, a recent taxonomy proposal delineated four new genera, designated *Alpha*-, *Beta*-, *Gamma*-, and *Delta*-polyomavirus (8). Recently, black sea bass-associated polyomavirus 1 (BassPyV1, GenBank accession number KP071318), the first polyomavirus associated with a bony fish (*Centropristis striata*), was described (9). Here, a complete polyomavirus genome is reported from a cartilaginous fish, the giant guitarfish (*Rhynchobatus djiddensis*), a batoid elasmobranch (order *Rajiformes*). The presence of proliferative skin lesions, characterized microscopically by large intranuclear inclusions containing 75 nm icosahedral viral particles, initiated an investigation of the causative agent (10). To circumvent the lack of known viral genetic information in elasmobranchs, a sequence-independent metagenomic approach was performed to identify viral sequences within the lesions (11, 12).

A complete, circular, double-stranded, 3,962 bp DNA genome was characterized. This virus, giant guitarfish polyomavirus 1 (GfPyV1), has characteristic polyomavirus arrangement of major open reading frames, including LT, VP1, and VP2. Although transmission electron microscopy failed to identify polyomavirus-like particles in tissue and virus isolation was not attempted due to lack of compatible cell lines, the presence of GfPyV1 LT and VP1 nucleic acids in skin lesions were confirmed using nested PCR and Sanger sequencing.

The genome size of 3.96 kb makes GfPyV1 the smallest described polyomavirus, compared with other genomes of 4.7 to 7.4 kb (2, 13, 14). While the GfPyV1 genome showed typical polyomavirus organization, its nucleotide sequence is highly divergent from other polyomaviruses. The predicted 1,794 bp large T (LT) protein is encoded by a single open reading frame, in contrast to the spliced LT genes of other polyomaviruses. BLAST searches revealed roughly 30% identity to a variety of mammalian and avian polyomavirus LT proteins. The LT from GfPyV1contains predicted DnaJ, Ori-binding, and helicase domains typical of polyomaviruses (15). A possible small T antigen-like open reading frame (ORF) encoding a 75 amino-acid-long protein was also predicted in the GfPyV1 early region, but a BLAST search revealed no sequence identity to any proteins in GenBank.

The predicted major capsid protein (VP1) contains 277 amino acids, smaller than all known VP1 proteins (2). It shares roughly 25% identity with various polyomavirus VP1 coat proteins by BLASTp search. At 500 amino acids, the predicted minor capsid protein (VP2) is longer than typical VP2 proteins (2). The VP2 encodes a possible N-terminal myristoylation signal.

Comparing the two fish polyomaviruses using Sequence Declamation Tool (SDT) v1.0 (16), BassPyV1 and GfPyV1 share 19.3%, 26%, 27.8%, and 22.9% protein identity in the viral genes LT, ST, VP1, and VP2, respectively. Although GfPyV1 DNA was present in associated tissues, preliminary data suggests that it was not the cause of the skin lesions.

### Nucleotide sequence accession numbers. 

The complete genomic sequence of guitarfish polyomavirus 1 was deposited in GenBank under the GenBank accession numbers NC_026244 and KP264963.
